# Eomes^hi^ NK Cells in Human Liver Are Long-Lived and Do Not Recirculate but Can Be Replenished from the Circulation

**DOI:** 10.4049/jimmunol.1601424

**Published:** 2016-10-21

**Authors:** Antonia O. Cuff, Francis P. Robertson, Kerstin A. Stegmann, Laura J. Pallett, Mala K. Maini, Brian R. Davidson, Victoria Male

**Affiliations:** * Division of Infection and Immunity, Institute of Immunity and Transplantation, University College London, London NW3 2PF, United Kingdom; and; † Department of Surgery and Interventional Science, University College London, Royal Free Hospital, London NW3 2QG, United Kingdom

## Abstract

Human liver contains an Eomes^hi^ population of NK cells that is not present in the blood. In this study, we show that these cells are characterized by a molecular signature that mediates their retention in the liver. By examining liver transplants where donors and recipients are HLA mismatched, we distinguish between donor liver–derived and recipient-derived leukocytes to show that Eomes^lo^ NK cells circulate freely whereas Eomes^hi^ NK cells are unable to leave the liver. Furthermore, Eomes^hi^ NK cells are retained in the liver for up to 13 y. Therefore, Eomes^hi^ NK cells are long-lived liver-resident cells. We go on to show that Eomes^hi^ NK cells can be recruited from the circulation during adult life and that circulating Eomes^lo^ NK cells are able to upregulate Eomes and molecules mediating liver retention under cytokine conditions similar to those in the liver. This suggests that circulating NK cells are a precursor of their liver-resident counterparts.

## Introduction

Natural killer cells are lymphocytes that were first identified by their ability to kill tumor cells without the need for prior sensitization. The best characterized NK cells develop in the bone marrow, circulate in the blood, and have a role in the immune defense against viruses and cancer. However, NK cells are also found in large numbers in nonlymphoid organs, including the uterus and liver (1). Organ-specific NK cells differ phenotypically from their circulating counterparts and are also likely to have specialist physiological functions relevant to their home organs (2). For example, uterine NK cells mediate placental implantation during pregnancy (3, 4).

Recently, NK cells in the liver have been a focus of intense research interest. In mice, splenic NK cells almost uniformly express the T-box transcription factor Eomes, but in the liver, a distinct population of Eomes^−^ NK cells is also present (5). These murine Eomes^−^ NK cells have an immature phenotype and were originally thought to be precursors to Eomes^+^ circulating NK cells (5). More recently, it has been proposed that Eomes^−^ liver NK cells form a separate lineage from Eomes^+^ circulating NK cells (2, 6). Suggestively, the transcription factors required for the development of the two NK cell subsets differ, with circulating NK cells requiring Eomes (5) and E4bp4 (2, 7, 8), whereas liver NK cells develop independently of these, but instead require T-bet (2, 5, 6). Furthermore, sorted Eomes-GFP^−^ liver NK cells are not able to differentiate into Eomes^+^ NK cells (6). Parabiosis experiments show that T-bet–dependent liver NK cells, defined in these studies as DX5^−^CD49a^+^, do not leave the liver, providing definitive evidence that these NK cells are liver resident (2, 9).

There have been three recent reports of NK cell subsets enriched in human liver, compared with blood, defined either as CD49a^+^ (10), CD56^bright^ (11), or CXCR6^+^ (12). The enrichment of these subsets in liver, and their expression of CD69, is suggestive of residency, but the difficulties of working with human subjects mean that definitive experiments to address whether these NK cells are liver resident have not yet been performed (13).

We previously postulated that human liver, similar to that of the mouse, might contain a liver-specific NK cell population defined by its lack of Eomes expression. Human liver does contain an NK cell population that is not present in blood but, in contrast to the liver-specific population in the mouse, it is Eomes^hi^ (12). In this study, we demonstrate that these cells express a signature of molecules that mediate their retention in the liver. Working with HLA-mismatched human liver transplants, we show that Eomes^hi^ NK cells are not able to exit the liver and are long-lived, capable of surviving in the liver for up to 13 y. This indicates that these are genuine liver-resident cells. Eomes^hi^ NK cells can be replenished from the circulation during adult life, and cytokines found at high concentrations in the liver promote the upregulation of Eomes. This suggests that, in humans, Eomes^lo^ circulating NK cells may be recruited to the liver where they upregulate Eomes becoming long-lived liver-resident cells.

## Materials and Methods

### Samples

Perfusion fluid was obtained from 16 healthy livers used for transplantation and 11 healthy livers that were unsuitable for transplantation due to vascular abnormalities, long warm ischemic time, or because of primary tumors found in other organs. Sixteen of the donors were male and 11 female with age range of 15–74 y (median, 42 y). Biopsies were taken from the explanted livers of five patients receiving their second liver transplant. Ethical approval for use of blood, perfusates, and explanted liver biopsies was obtained through the Royal Free Hospital Biobank (National Health Service Research Ethics Committee approval no. 11/WA/0077, study no. 9455). Pre- and postimplant biopsies were collected as part of the RIPCOLT trial (National Health Service Research Ethics Committee approval no. 11/H0720/4, trial number 8191).

Leukocytes from perfusion fluid were concentrated by centrifugation (750 × *g*, 15 min, 20°C). The concentrated cells were layered onto Ficoll (GE Healthcare, Amersham, U.K.), centrifuged (400 × *g*, 20 min, 20°C, light braking), and the interface was taken and washed twice with PBS (750 × *g*, 15 min, 20°C). Tissue from explanted livers were finely minced using scalpels, passed through a 70-μm strainer, and the collected cells were layered onto Ficoll, centrifuged (400 × *g*, 20 min, 20°C, light braking), and the interface was taken and washed twice with PBS (750 × *g*, 15 min, 20°C). Trucut biopsies were pushed through a 40-μm strainer and the cell suspension was used without further purification.

### Flow cytometry

The following Abs were used: from eBioscience (San Diego, CA): CD3-allophycocyanin eFluor 780 (clone SK7), CD16-FITC (eBioCB16), CD19-allophycocyanin eFluor 780 (HIB19), CD45-PE (HI30), CD94-FITC (DX22), Eomes-PE eFluor 610 (WD1928), granzyme K–PerCP eFluor 710 (G3H69), HLA-A3-FITC (GAP.A3), IFN-γ–Alexa Fluor 488 (4S.B3), S1PR1–eFluor 660 (SW4GYPP), T-bet–PE-Cy7 (4B10), and TNF-α–allophycocyanin (Mab11); from BioLegend (London, U.K.): CCR5-allophycocyanin (J418F1), CD49a-FITC (TS2/7), CD69-allophycocyanin (FN50), CD103-FITC (Ber-ACT8), CX3CR1-FITC (2A9-1), CXCR6-PerCP Cy5.5 (K041E5), CXCR6-allophycocyanin (K041E5), GM-CSF–PE (BDV-21C11), granzyme B–FITC (GB11), HLA-A2–FITC (BB7.2), KIR2DL1/S1/S3/S5-allophycocyanin (HP-MA4), KIR2DL2/L3-allophycocyanin (DX27), KIR3DL1-allophycocyanin (DX9), and perforin-allophycocyanin (dG9); and from BD Biosciences (Oxford, U.K.): CD56-BV510 (NCAM16.2) and LIF-PE (1F10). Dead cells were excluded using fixable viability dye eFluor 450 (eBioscience). Intracellular staining was carried out using Human FoxP3 Buffer (BD Biosciences) according to the manufacturer’s instructions. Data were acquired on an LSRFortessa II (BD Biosciences) and analyzed using FlowJo (Tree Star, Ashland, OR). Cells were sorted on a FACSAria (BD Biosciences). Eomes^lo^ NK cells were isolated by sorting on live cells (propidium iodide^−^, Tonbo Biosciences, San Diego, CA), singlets, scatter, and CD3^−^CD56^+^CXCR6^−^CD16^+^. Eomes^hi^ NK cells were isolated by sorting on live cells, singlets, scatter, and CD3^−^CD56^+^CXCR6^+^.

### RNA sequencing

Total RNA was extracted from sorted cells using an RNeasy Micro kit (Qiagen, Manchester, U.K.), and cDNA was amplified using a SMART-Seq ultra-low input RNA kit for sequencing (Takara Bio Europe/Clontech, Saint-Germain-en-Laye, France). Amplified cDNA (200 pg) was used as input for library preparation using a Nextera XT DNA library preparation kit (Illumina, Essex, U.K.) with 12 cycles of PCR. Samples were sequenced on a NextSeq 500 (Illumina). Alignments were performed using TopHat, and regularized log-transformed normalization was performed using DESeq2 (BaseSpace; Illumina). Expression of each gene was compared using a paired *t* test. Further analysis was undertaken by Ingenuity Pathway Analysis (Qiagen) with a fold change cutoff of 2 and a significance cutoff of 0.01.

### Functional assays

Sorted cells were cultured with K562 for 4 h at a 1:1 ratio in 50 μl of RPMI 1640 medium supplemented with 10% FCS, 25 mM HEPES, 1 mM sodium pyruvate, 50 μM 2-ME, MEM nonessential amino acids, penicillin, and streptomycin (all Life Technologies brand; Thermo Fisher Scientific, Hudson, NH). Cells were harvested and stained with Annexin V^FITC^ (BD Biosciences) and propidium iodide.

Freshly isolated perfusate cells (10^6^) were stimulated with PMA (25 ng/ml; Sigma-Aldrich, Hammerhill, U.K.) and ionomycin (1 μg/ml; Sigma-Aldrich) for 4 h with brefeldin A (10 μg/ml; Sigma-Aldrich), monensin (2 μM; Sigma-Aldrich), and 5 ng/ml PerCP–eFluor 710-conjugated anti-human CD107a (clone eBioH4A3; eBioscience). Intracellular staining for cytokines was performed at the end of the assay.

### In vitro differentiation assays

Cells were plated at 2.5 × 10^4^/ml in RPMI 1640 medium, supplemented as before, and with 5 ng/ml recombinant human IL-7 (PeproTech, Rocky Hill, NJ). Recombinant human IL-15 (20 ng/ml), IL-12 (50 ng/ml), or TGF-β (5 ng/ml) (all PeproTech) was added. Cells were cultured for 7 d with a half medium change at days 2 and 5.

### NKL transduction

Lentivirus was produced by transfecting 293T cells with pCSGW-IRES-dsRed (vector) or pCSGW-Eomes-IRES-dsRed and the packaging plasmids psPAX2 and pMD2.G (deposited at Addgene as plasmids p12259 and p12260, respectively, by D. Trono, École Polytechnique Fédérale de Lausanne, Lausanne, Switzerland). NKL cells were transduced by spinfection at 700 × *g* and 20°C for 45 min with 10 μg/ml Polybrene (Sigma-Aldrich) and cultured for a further 72 h before harvesting and examination.

## Results

### Eomes^hi^ NK cells are present in human liver but not in blood and have a distinct phenotype

**FIGURE 1.**
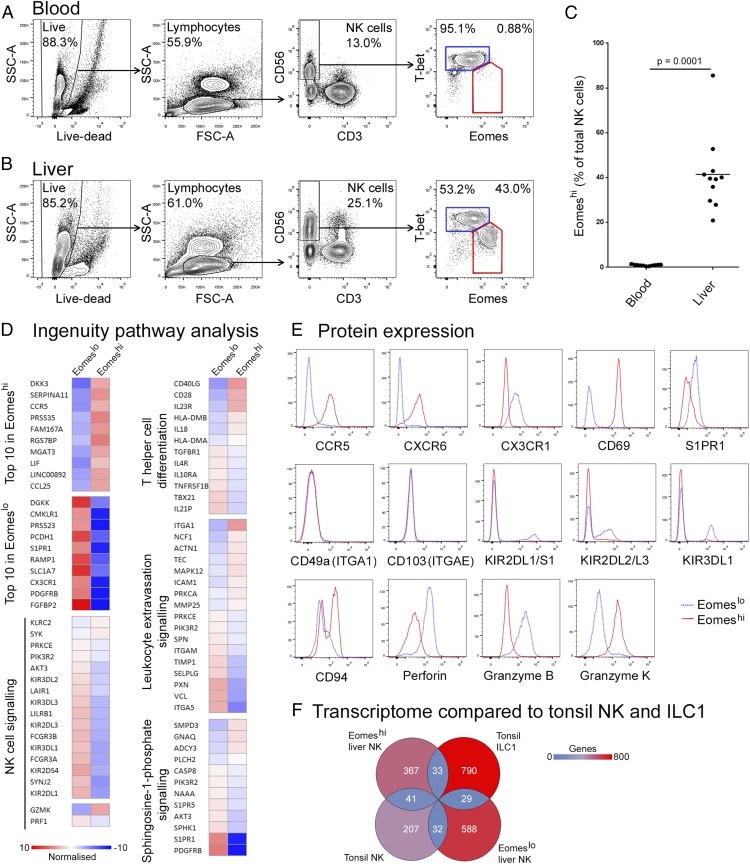
Eomes^hi^ NK cells are present in human liver and have a distinct phenotype. (**A**) PBLs were isolated from healthy volunteers and NK cells identified by gating on live cells, scatter, CD3^−^, and CD56^+^. NK cells were examined for their expression of Eomes and T-bet. (**B**) Leukocytes were isolated from the perfusion fluid of healthy human livers destined for transplantation and stained as in (A). (**C**) Summary data showing Eomes^hi^ NK cells as a percentage of total NK cells across 11 samples. Significance was determined using a Mann–Whitney *U* test. (**D**) RNASeq data from Eomes^lo^ and Eomes^hi^ NK cells sorted from the perfusion fluid from *n* = 5 healthy livers. Differentially expressed genes were identified using paired *t* tests. The top 10 most differentially expressed genes and the top most significantly enriched canonical pathways are shown. (**E**) Flow cytometry for key proteins expected to differ between Eomes^lo^ (blue dashed line) and Eomes^hi^ (red solid line) liver NK cells. Histograms are representative of *n* = 4 independent samples. (**F**) Overlap between top upregulated genes in Eomes^lo^ and Eomes^hi^ liver NK cells, compared with tonsil NK cells and ILC1 (23).

Prior to transplantation, donor livers are perfused with cold University of Wisconsin fluid. The perfusion fluid (perfusate) contains large numbers of leukocytes with a composition that mirrors that found in biopsies (11, 12, 14). Examining perfusates from 11 transplanted livers, we confirmed our previous findings that both Eomes^lo^ and Eomes^hi^ NK cells were present in human liver, whereas circulating NK cells were uniformly Eomes^lo^ (Fig. 1A–C) (12).

We postulated that these Eomes^hi^ liver-specific NK cells might have a specialist physiological function. To take an unbiased approach to this question, we sorted Eomes^lo^ and Eomes^hi^ NK cells from five perfusates and performed RNA sequencing (RNAseq) analysis (the RNAseq data and differentially expressed gene list were deposited at National Center for Biotechnology Information Gene Expression Omnibus under accession no. GSE87392; https://www.ncbi.nlm.nih.gov/geo/query/acc.cgi?acc=GSE87392). Ingenuity Pathway Analysis (Fig. 1D) identified the two most significantly enriched canonical pathways as NK cell signaling (*p* = 1.12 × 10^−6^) and Th cell signaling (*p* = 1.10 × 10^−5^), although neither of these was identified as being more highly activated in either NK cell subset. Among the 15 most significantly enriched canonical pathways, leukocyte extravasation signaling was the most highly activated in Eomes^hi^ NK cells (*p* = 1.74 × 10^−3^; *z*-score = 1.528), whereas sphingosine-1-phosphate signaling was the most highly activated in Eomes^lo^ NK cells (*p* = 1.17 × 10^−3^; *z*-score = −1.667). Other canonical pathways in the top 15 were mostly those whose component genes overlap with these pathways, for example ILK signaling (large overlap with leukocyte extravasation signaling) and ceramide signaling (large overlap with sphingosine-1-phosphate signaling). The top upstream regulator was TGF-β1 (*p* = 4.01 × 10^−15^).

We next examined protein expression of some genes identified as differing at the transcript level (Fig. 1E). Eomes^hi^ NK cells expressed higher levels of CXCR6 and CCR5 than did Eomes^lo^ NK cells, but lower levels of CX3CR1, in agreement with previous observations on bulk liver NK cells compared with blood (11) and similar to NK cells enriched in lymphoid organs (15). S1PR1, which mediates migration of immune cells from tissues into the circulation (16), was expressed at lower levels on Eomes^hi^ NK cells, whereas CD69, which negatively regulates S1PR1, was expressed at higher levels, similar to findings in other subsets of NK cells proposed to be resident in the liver or lymphoid organs (10, 11, 15). ITGA1, which encodes the key marker of liver residency in mice, integrin α_1_ (or CD49a) (2, 9), and has been proposed to define liver-resident NK cells in humans (10) was highly overexpressed in Eomes^hi^ NK cells at the transcript level, but we were unable to detect any difference in protein expression. ITGAE encodes integrin α_E_, or CD103, a marker of residence in CD8^+^ memory T cells (17), and it was also overexpressed by Eomes^hi^ NK cells at the transcript level, but again we were unable to detect a difference at the protein level.

In agreement with the RNAseq data, Eomes^hi^ NK cells express killer cell Ig-related receptors at a far lower frequency than do Eomes^lo^ NK cells, but CD94 (coreceptor to NKG2A, or KLRC2) is expressed at a higher frequency. Eomes^hi^ NK cells also express lower levels of perforin and granzyme B but higher levels of granzyme K. The decreased expression of proteins associated with cytotoxicity on Eomes^hi^ NK cells might suggest that they are less cytotoxic than their Eomes^lo^ counterparts. Indeed, Eomes^hi^ NK cells were somewhat less able to kill K562 target cells than were Eomes^lo^ NK cells, although both did have some cytotoxic capacity (Supplemental Fig. 1A). Perhaps surprisingly, given their lesser ability to kill target cells, it was the Eomes^hi^ NK cells that had the greater propensity for degranulation (Supplemental Fig. 1B). We assessed IFN-γ, TNF-α, and GM-CSF production (Supplemental Fig. 1C–E), because these have been shown to differ between Eomes^−^ and Eomes^+^ NK cells in mice (2, 5, 6, 9, 18). Among these, the only cytokine to differ between the subsets was TNF-α, which was produced at somewhat lower levels by Eomes^hi^ NK cells. Finally, we assessed the ability of the cells to produce LIF, because this was one of the most upregulated genes in Eomes^hi^ NK cells by RNAseq, and IL-22, because IL-23R was also overexpressed in Eomes^hi^ NK cells. However, under the conditions examined, both subsets made very little of either of these cytokines and there was no significant difference between them (Supplemental Fig. 1F, 1G). We undertook all these experiments under a variety of conditions, including coculture with K562, culture with IL-12 alone, culture with IL-12 and IL-18, and (for IL-22 production) 4-h culture with IL-1β and IL-23 and overnight culture with IL-1β and IL-23, followed by restimulation with PMA and ionomycin. In all cases the results were similar to 4-h culture with PMA and ionomycin (Supplemental Fig. 1B–G).

### Neither liver NK cell subset resembles innate lymphoid cell types 1 or 3

In light of suggestions that liver-resident Eomes^−^ NK cells in mice represent innate lymphoid cell (ILC)1, whereas the Eomes^+^ population represents conventional NK cells (19), we sought to assess whether either liver NK cell subset found in humans closely resembled ILC1. Two ILC1 populations have been defined in human lymph nodes: a lineage^−^ (CD56^−^)CD127^+^CRTH2^+^c-Kit^−^NKp44^−^ population (20) and a CD56^+^NKp44^+^CD103^+^ population (21). Neither liver NK cell population is phenotypically identical to these, because they are both CD56^+^ and CD103^−^ (Fig. 1B, 1E). However, ILC1 notoriously varies in its phenotype between organs (22), so this does not rule out that one of these subsets might represent ILC1. Therefore, we used data on the whole transcriptomes of ILC1 and NK cells sorted from human lymph nodes (23) to generate a list of differentially expressed genes using the R package SCDE (24). Comparing this to the genes differentially expressed between Eomes^lo^ and Eomes^hi^ liver NK cells revealed that each NK subset shared roughly the same proportion of genes with both conventional NK cells and ILC1 isolated from the tonsils (Fig. 1F). We also considered the possibility that one of the liver NK cell subsets might represent a CD56^+^ ILC3 population. However, neither NK subset produced significant IL-22 with any of the stimulation regimes we tried (Supplemental Fig. 1G). Therefore, we conclude that both the Eomes^lo^ and the Eomes^hi^ CD3^−^CD56^+^ subsets present in the liver are likely to represent NK cells, and not helper ILCs.

### Eomes^hi^ NK cells do not exit the liver and are long-lived in the liver

The findings that Eomes^hi^ NK cells were present in liver but not blood, and that the most altered pathways in Eomes^hi^ NK cells are associated with blood exit and tissue retention, suggested that Eomes^hi^ NK cells might represent a liver-resident population. To investigate this possibility, we designed experiments to assess whether Eomes^hi^ NK cells are capable of leaving the liver and whether they are long-lived in the liver.

**FIGURE 2.**
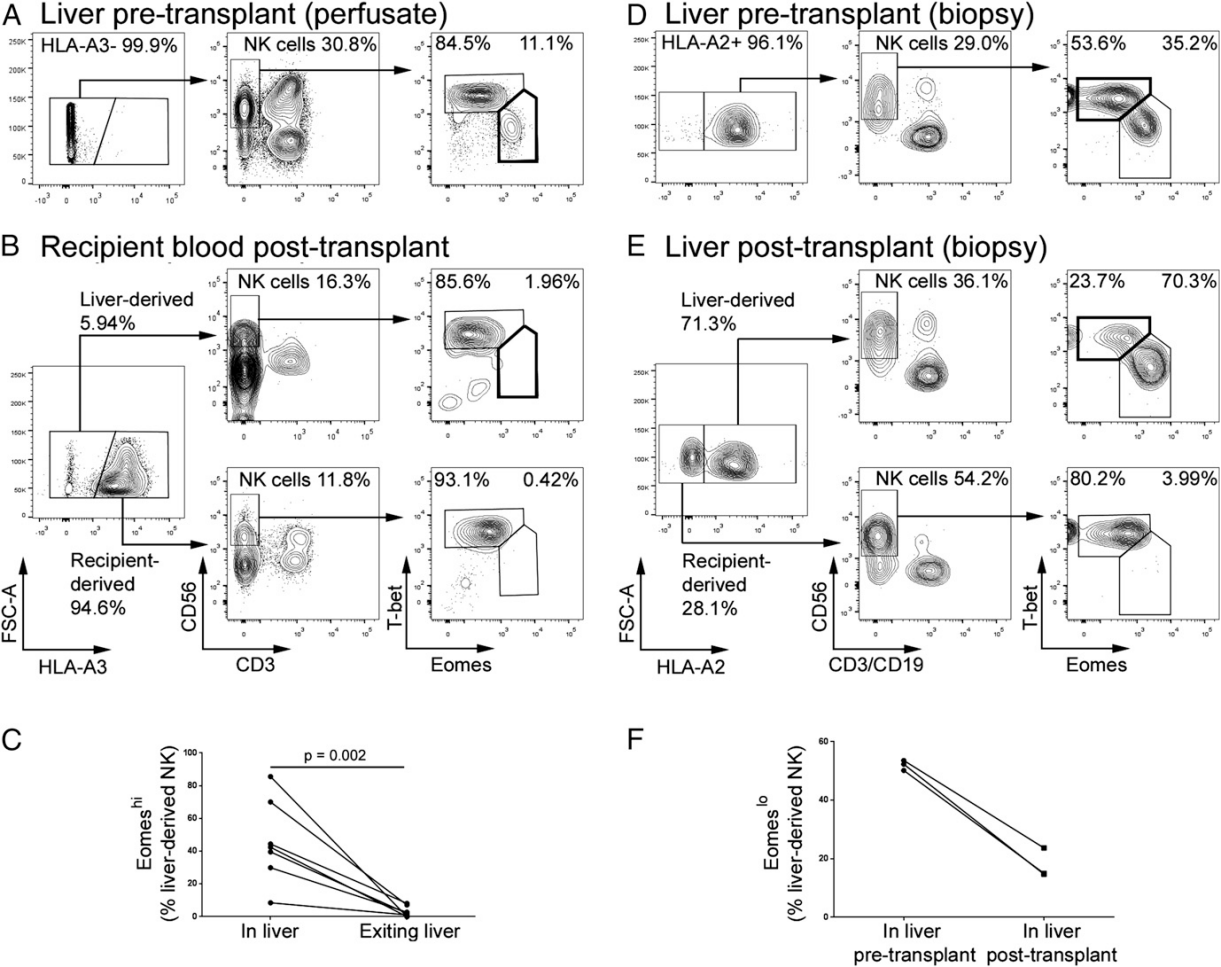
Eomes^lo^ NK cells exit the liver but Eomes^hi^ NK cells do not. (**A** and **B**) Example of staining from a single transplant in which an HLA-A3–negative liver (A) was transplanted into an HLA-A3–positive recipient. Twenty-four hours after transplant, PBLs were isolated from the recipient’s blood and circulating liver-derived cells were distinguished from recipient-derived cells on the basis of HLA-A3 staining (B). NK cells were examined for their expression of Eomes and T-bet. (**C**) Summary data from *n* = 7 transplants mismatched at either HLA-A2 or HLA-A3. Significance was determined using a paired *t* test. (**D** and **E**) Example staining from a single transplant in which an HLA-A2–positive liver (D) was transplanted into an HLA-A2–negative recipient. Three hours posttransplant, a second biopsy was taken from the liver and liver-derived cells were distinguished from recipient-derived cells on the basis of HLA-A2 staining (E). (**F**) Summary data from *n* = 3 transplants, all mismatched at HLA-A2. The gates used to produce the summary data (C and F) are highlighted.

We identified seven liver transplants in which the donor and recipient were mismatched for either HLA-A2 or HLA-A3. In these transplants, we could distinguish between donor liver–derived and recipient leukocytes by staining with Abs specific for the mismatched HLA. We examined the composition of leukocytes in donor liver perfusate before the transplant and in recipient blood 24–48 h after the transplant (clinical details are given in Supplemental Table I). As expected, before transplantation both Eomes^lo^ and Eomes^hi^ NK cells were present in donor livers (Fig. 2A, 2C), and only Eomes^lo^ NK cells were present in recipient blood. After transplant, we were able to identify cells in the recipient’s blood that had recently exited the donor liver. NK cells were among these but were uniformly Eomes^lo^ (Fig. 2B, 2C), indicating that only Eomes^lo^ NK cells are able to leave the liver. We also examined three paired pretransplant and 3 h posttransplant liver biopsies (Fig. 2D–F). The proportion of donor liver–derived Eomes^lo^ NK cells decreased after transplant, in support of the idea that Eomes^lo^ NK cells are able to leave the liver. Taken together, these findings indicate that Eomes^lo^ NK cells recirculate whereas Eomes^hi^ NK cells do not, consistent with the hypothesis that Eomes^hi^ NK cells are liver resident.

Another hallmark of residence is longevity. To determine whether Eomes^hi^ NK cells are long-lived in the liver, we identified five patients who had previously received a liver transplant mismatched for either HLA-A2 or HLA-A3 and were now receiving their second transplant (clinical details are given in Supplemental Table I). We isolated leukocytes from the explanted first transplant and distinguished between donor liver–derived and recipient-derived cells to determine which leukocytes were able to survive in the liver without replenishment from the circulation. One important caveat is that most of the patients receiving their second transplant were already undergoing immunosuppressive therapy following their first transplant, or they were suffering from immune-mediated complications, or both. However, we did find that the proportions of T cells, NK cells, and Eomes^lo^ and Eomes^hi^ cells as a proportion of total NK cells were not significantly different between healthy livers, disease controls receiving their first transplant, and our cohort of five retransplant patients, suggesting that our findings are likely to be broadly representative of the situation in healthy liver and other liver diseases (Supplemental Fig. 2).

**FIGURE 3.**
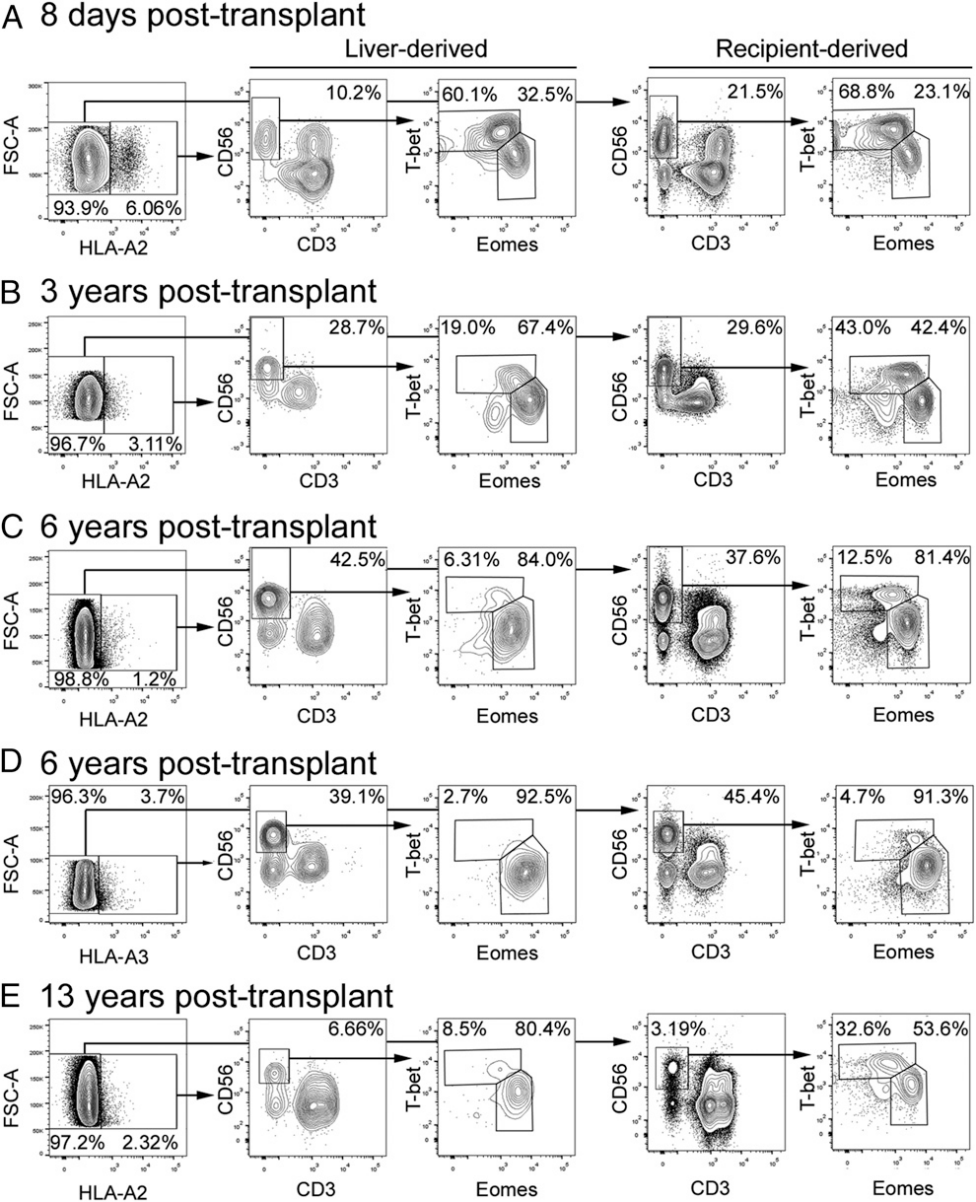
Eomes^hi^ NK cells are long-lived in the liver, but they can be recruited from the circulation during adult life. (**A**) An HLA-A2–positive liver was transplanted into an HLA-A2–negative recipient and removed 8 d later. Liver leukocytes were isolated from a biopsy and liver-derived cells were distinguished from recipient-derived cells on the basis of HLA-A2 staining. Liver- and recipient-derived NK cells present in the liver were examined for their expression of Eomes and T-bet. (**B**) An HLA-A2–positive liver was transplanted into an HLA-A2–negative recipient and removed 3 y later. (**C**) An HLA-A2–positive liver was transplanted into an HLA-A2–negative recipient and removed 6 y later. (**D**) An HLA-A3–positive liver was transplanted into an HLA-A3–negative recipient and removed 6 y later. (**E**) An HLA-A2–positive liver was transplanted into an HLA-A2–negative recipient and removed 13 y later.

Eomes^lo^ NK cells retained in the liver were present in the three biopsies taken immediately after transplant (Fig. 2E, 2F) and in livers explanted 8 d and even 3 y posttransplant (Fig. 3A, 3B), but they were barely detectable in livers collected ≥6 y posttransplant (Fig. 3C–E). This is consistent with Eomes^lo^ NK cells continually leaving the liver and being replaced from the circulation. In contrast, liver-derived Eomes^hi^ NK cells were present in all livers examined (Fig. 3A–E), persisting in small numbers even in the liver collected 13 y posttransplant. This suggests that the Eomes^hi^ NK cell population is indeed long-lived and supports the view that it is liver resident.

### Eomes^hi^ liver NK cells can be recruited from the circulation

Our examination of recipient-derived cells isolated from posttransplant biopsies showed that Eomes^lo^ NK cells are rapidly recruited from the circulation (Fig. 2E). At this early time, no recipient-derived Eomes^hi^ NK cells are present. This is unsurprising, because Eomes^hi^ NK cells do not circulate. However, when we examined the recipient-derived cells present in explanted livers (Fig. 3) we found Eomes^lo^ circulating NK cells, as expected, but also Eomes^hi^ NK cells, present even as early as 8 d posttransplant (Fig. 3A). This suggested that some precursor of the Eomes^hi^ NK cells could be recruited to the liver from the circulation. Given that circulating Eomes^lo^ NK cells are rapidly recruited to the liver (Fig. 2E), we investigated the possibility that these could be precursors of Eomes^hi^ liver-resident NK cells.

### Eomes^lo^ NK cells can upregulate Eomes

We first sought to determine whether Eomes^lo^ and Eomes^hi^ NK cells in the liver form separate lineages, as is thought to be the case in mice (6). We therefore sorted the two NK cell populations and cultured them for 7 d to assess their ability to cross-differentiate. We included IL-15 in all culture conditions because it is required for NK cell development and survival (25, 26). TGF-β was included in one condition because it was identified as a top upstream regulator in our RNAseq experiment, it is highly expressed in the liver (27), and it promotes residence in CD8^+^ memory T cells (17, 28). This condition might be expected to move Eomes^−^ NK cells toward Eomes expression. IL-12 was included in another condition because it promotes T-bet expression (29–31) and T-bet negatively regulates Eomes (32, 33), so this condition might be expected to move Eomes^hi^ (T-bet^lo^) NK cells toward an Eomes^lo^ (T-bet^hi^) phenotype.

**FIGURE 4.**
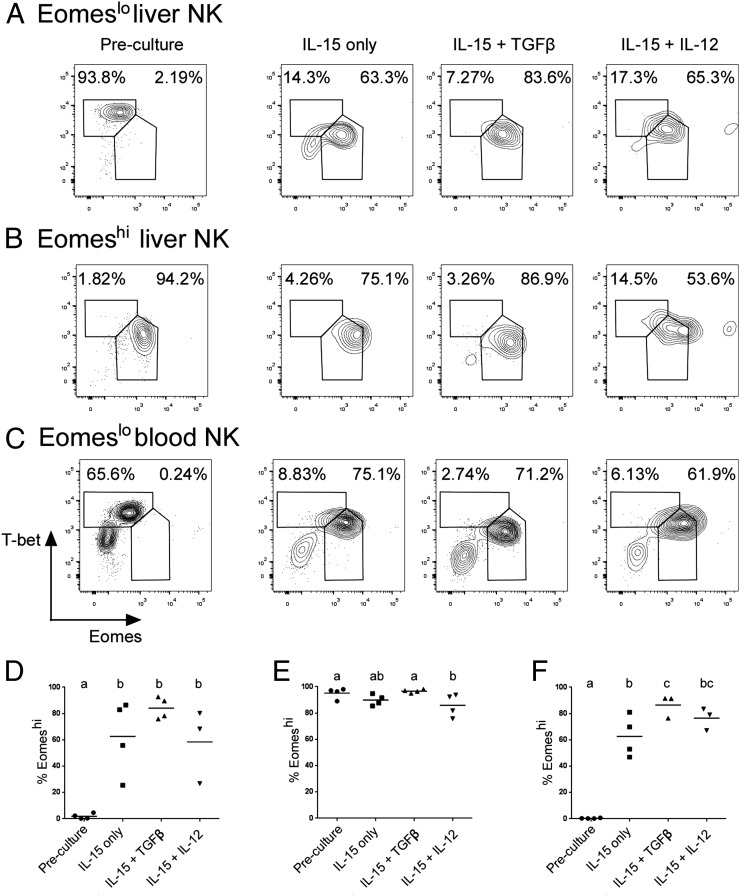
Eomes^lo^ NK cells can become Eomes^hi^. (**A** and **B**) NK cells were sorted from perfusion fluid [(A), Eomes^lo^; (B), Eomes^hi^)] and cultured for 7 d in the indicated conditions. At the end of the culture period, the cells were examined for their expression of Eomes and T-bet. (**C**) Sorted blood NK cells were cultured as above. (**D**–**F**) Summary data showing the percentage of Eomes^hi^ NK cells in *n* = 4 independent experiments, starting with Eomes^lo^ liver NK cells (D), Eomes^hi^ liver NK cells (E), or Eomes^lo^ peripheral blood NK cells (F). Groups that are significantly different (*p* < 0.05 by one-way ANOVA) are indicated by different letters.

**FIGURE 5.**
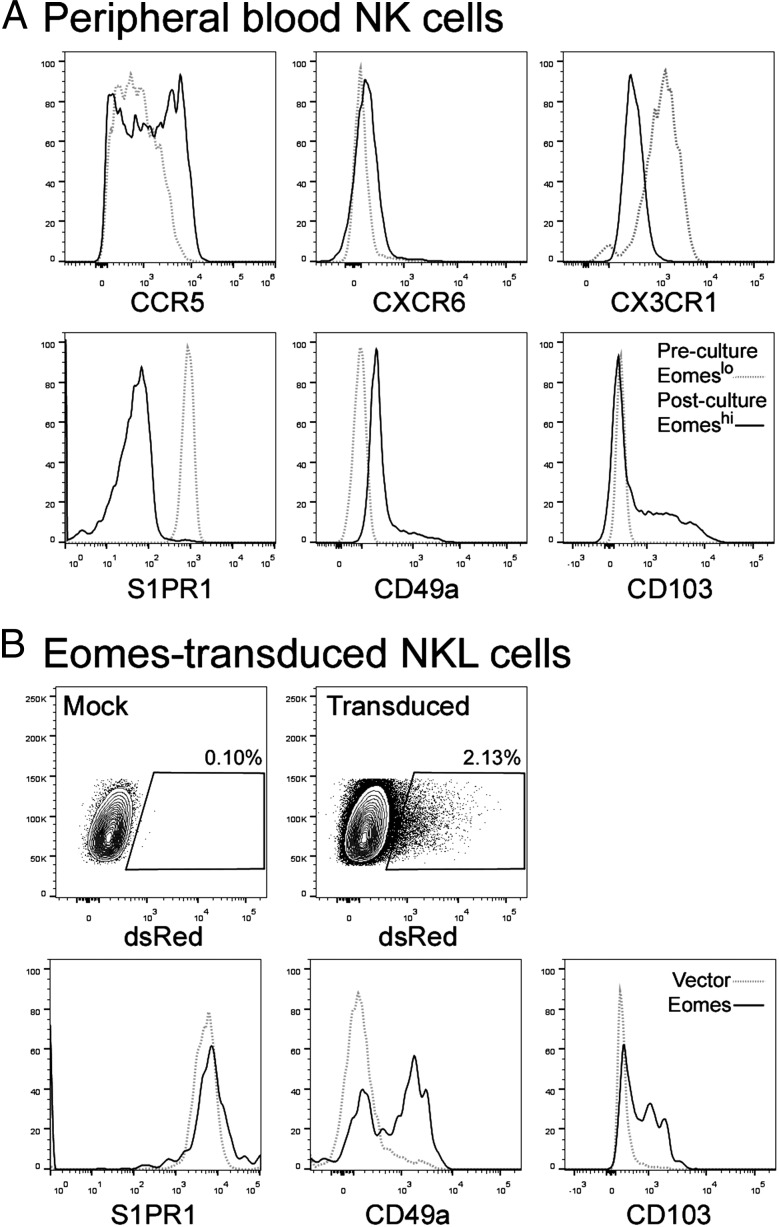
Eomes upregulation is associated with increased expression of mediators of tissue retention. (**A**) Sorted blood NK cells were cultured for 7 d in IL-15 and TGF-β to produce in vitro–derived Eomes^hi^ NK cells. Eomes^lo^ NK cell expression of the indicated proteins before culture (gray dashed line) and Eomes^hi^ NK cell expression after culture (black solid line) is shown. (**B**) NKL cells were transduced with Eomes or vector control and gated on transduced (dsRed^+^) cells. Expression of the indicated proteins in vector-transduced cells (gray dashed line) and Eomes-transduced cells (black solid line) is shown. The histograms show a single experiment, representative of *n* = 4.

Under all these conditions, Eomes^lo^ liver NK cells upregulated Eomes and downregulated T-bet (Fig. 4A, 4D). We were less successful at causing Eomes^hi^ NK cells to downregulate Eomes, even on culture with high concentrations of IL-12 (Fig. 4B, 4E), although under these conditions they did somewhat increase their expression of T-bet. In vitro–differentiated NK cells did not alter their expression of CXCR6 (Fig. 5A), confirming that the Eomes^lo^ NK cells increased Eomes expression, rather than the observations resulting from outgrowth of contaminating Eomes^hi^ (CXCR6^+^) cells.

Having shown that Eomes^lo^ NK cells in the liver can become Eomes^hi^, we sought to determine whether the same is also true of Eomes^lo^ peripheral blood NK cells. Similar to Eomes^lo^ liver NK cells, sorted CD3^−^CD56^+^ blood NK cells upregulated Eomes in culture (Fig. 4C, 4F), supporting the hypothesis that circulating NK cells could, under the influence of cytokines that are highly expressed in the liver, acquire Eomes expression.

### Eomes upregulation is associated with increased expression of mediators of tissue retention

If circulating NK cells are recruited to the liver, upregulating Eomes and concomitantly becoming liver resident, it seems likely that Eomes is causing altered expression of chemokine receptors, integrins, and S1PR1, which results in the cells being retained in the liver. To further investigate this, we examined cell surface expression of CXCR6, CCR5, CX3CR1, CD49a, CD103, and S1PR1 in freshly isolated Eomes^lo^ peripheral blood NK cells and peripheral blood NK cells that had been cultured for 7 d in IL-15 and TGF-β to promote the upregulation of Eomes (Fig. 5A). We found no change in the expression of CXCR6 during culture, but we did find that CCR5 expression increased whereas CX3CR1 and S1PR1 decreased, consistent with Eomes^hi^ liver NK cells isolated ex vivo. We also found that the expression of CD49a and CD103 increased in in vitro–differentiated Eomes^hi^ NK cells, consistent with the increased expression of ITGA1 (CD49a) and ITGAE (CD103) mRNA in Eomes^hi^ NK cells isolated ex vivo.

These observations suggested that Eomes might directly alter the expression of genes that act to retain NK cells in the liver. However, it is also possible that the alterations we observed were caused by other aspects of the culture conditions, rather than being Eomes mediated. To more precisely define which genes were altered specifically as a result of Eomes expression, we lentivirally transduced NKL cells with either empty vector or Eomes and examined transduced cells (Fig. 5B). NKL cells are derived from a human NK cell leukemia (34). We used this cell line because it is more easily transduced than primary peripheral blood NK cells and yet is identical to them with respect to expression of Eomes, CD49a, CD103, and S1PR1. Unlike peripheral blood NK cells (but similar to other NK cell lines), NKL cells express intermediate levels of CCR5, CXCR6, and CX3CR1, so we did not examine alterations in these chemokine receptors. Eomes-transduced NKL cells expressed higher levels of CD49a and CD103 than did control-transduced cells (Fig. 5B), suggesting that the increase in the expression of these integrins in in vitro–differentiated Eomes^hi^ NK cells may be a direct result of Eomes action. In contrast, S1PR1 expression did not differ, indicating that decreased S1PR1 expression in both ex vivo–isolated and in vitro–differentiated Eomes^hi^ NK cells may not be a direct result of the action of Eomes.

## Discussion

Recent years have seen an explosion in interest in liver-specific NK cells and particularly in the idea that there may be a liver-resident subset of NK cells. In mice, these liver-resident NK cells have been characterized in detail. They are dependent on T-bet but independent of Eomes and, using parabiosis experiments, have been definitively shown not to recirculate (2, 5, 6, 9). We took a comparable approach to identify liver-resident NK cells in humans. Similar to mouse liver, human liver contains both Eomes^lo^ and Eomes^hi^ NK cell subsets but, in contrast to the mouse, the Eomes^lo^ NK subset is present in both blood and liver whereas the Eomes^hi^ subset is restricted to the liver. The transcription factors regulating circulating versus liver-resident NK cells, therefore, seem to be reversed in humans compared with mice. This is consistent with a report of an infant with a silencing mutation in Eomes, who presented with a normal distribution of circulating NK cells, suggesting that Eomes is not required for their development in humans (35). That these two T-box transcription factors could have switched roles over the course of evolution should perhaps not be surprising in the light of their highly homologous nature and often redundant roles (36, 37).

By RNAseq, the most altered canonical pathways between the two liver NK cell subsets were associated with blood exit and tissue retention, and this suggests that the main difference between Eomes^lo^ and Eomes^hi^ NK cells is that the Eomes^hi^ population is liver resident. An earlier study showed that some NK cells can be retained in the liver for up to 2 y (38). We have extended this work by showing that some NK cells are retained for up to 13 y and that these long-term resident NK cells are the Eomes^hi^ population. This demonstration that Eomes^hi^ NK cells are unable to re-enter the circulation and are long-lived in the liver provides, to our knowledge for the first time, evidence of a bona fide liver-resident NK cell population in humans. To our knowledge, this is also the first such demonstration of a resident NK cell population in any human tissue.

Examination of recipient-derived cells from transplanted livers removed between 8 d and 13 y posttransplant further revealed that Eomes^hi^ liver-resident NK cells can be recruited from the circulation during adult life. Eomes^lo^ NK cells can become Eomes^hi^ when exposed to IL-15 and TGF-β, which are highly expressed in the liver (27, 39). This suggests that one source of Eomes^hi^ liver NK cells is circulating Eomes^lo^ NK cells, although these experiments do not rule out the possibility that Eomes^hi^ liver NK cells also derive, at least in part, from circulating CD34^+^ hematopoietic stem cells or NK progenitor cells (38, 40). There have been two recent reports of a small population of CXCR6^+^ NK cells in the blood, although these, unlike CXCR6^+^ NK cells in the liver, are Eomes^lo^ (12, 15). One possibility, then, is that circulating CXCR6^+^Eomes^lo^ NK cells are recruited to the liver by CXCL16, which is highly expressed by liver sinusoidal endothelial cells (11, 41) and there upregulate Eomes.

Our finding that Eomes^lo^ NK cells could give rise to Eomes^hi^ NK cells was unexpected in the light of work in mice, showing that liver-resident and circulating NK cells form separate lineages (6). It is, of course, likely that human and mouse NK cells differ in this respect, as they do in so many others. However, note that sorted circulating-type NK cells in mice can give rise to a small number of liver-type NK cells during 2 wk in vivo, so there may be some degree of flexibility between the lineages, even in mice (6). Furthermore, when bone marrow is transferred to a lethally irradiated mouse, a small proportion of donor-derived liver-resident NK cells are present 3 mo later (9), and when ILC progenitors are transferred to a Rag/γc double knockout host, donor-derived liver-specific NK cells are present after 3–6 wk (42). Therefore in mice, as we show in humans, there may also be some replacement of liver-resident NK cells from the circulation during adult life.

In vitro–differentiated Eomes^hi^ NK cells mirrored their ex vivo–isolated counterparts in their expression of CCR5, CX3CR1, and S1PR1. However, they did not express increased CXCR6. This is consistent with the proposition that CXCR6 causes recruitment to the liver where the cytokine environment promotes Eomes upregulation, as opposed to the alternative that Eomes causes CXCR6 expression. We also found increased expression of CD49a and CD103, strikingly similar to recent findings that TGF-β causes the upregulation of these integrins in circulating NK cells recruited to the salivary gland in the mouse (43). This is also in line with mRNA expression in freshly isolated Eomes^hi^ NK cells, although we did not detect a difference in protein expression ex vivo. It is possible that ex vivo–isolated Eomes^hi^ NK cells do express these proteins but at subdetectable levels, or that they express mRNA and are poised to produce protein but only do so under particular conditions. Transduction of NKL cells with Eomes also caused an increase in the expression of these integrins but did not alter expression of S1PR1. This could indicate that IL-15 and TGF-β alter S1PR1 via a parallel, Eomes-independent pathway, but could also potentially be a result of differences between the NKL cell line and primary NK cells. Nonetheless, it seems clear that IL-15 and TGF-β can cause peripheral blood NK cells to upregulate Eomes and concomitantly alter their expression of chemokine receptors, integrins, and S1PR1 in such a way as to promote retention in the liver, and that the alteration in integrin expression, at least, is likely to be a direct consequence of Eomes expression.

We therefore propose that CXCR6^+^Eomes^lo^ circulating NK cells are recruited to the liver by CXCL16 (12, 15, 41), where they are exposed to high concentrations of IL-15 and TGF-β (27, 39) causing the upregulation of Eomes. This in turn alters cell surface expression of chemokine receptors, integrins, and S1PR1 such that the Eomes^hi^ NK cells become unable to leave the liver and turn into long-lived resident cells. The precise function of these cells remains to be defined but, as has been suggested in the mouse, their residency may point to tissue-specific homeostatic functions (2). There is also some evidence that liver-resident NK cells in the mouse are memory cells (9, 44). The longevity of Eomes^hi^ NK cells in human liver could point to these cells also having memory, although this is an idea that will be challenging to test in humans.

The distinction between circulating and liver-resident NK cells in humans may also prove clinically relevant. Recently, evidence has emerged that the main drivers of ischemia-reperfusion injury in mouse kidney are resident NK cells (45). If the same proves to be true of human liver, this could suggest that targeting these cells in the donor liver prior to implantation may present a novel target to ameliorate ischemia-reperfusion injury in the setting of liver transplantation. Finally, the finding that a large proportion of the NK cells present in organs are likely to be resident suggests that we should reconsider the tendency to assume that observations made on NK cells circulating in the blood necessarily signify anything about their organ-resident counterparts (46, 47).

## Supplementary Material

Data Supplement
