# Supplementary material for: Eomeshi NK Cells in Human Liver Are Long-Lived and Do Not Recirculate but Can Be Replenished from the Circulation
Source: J Immunol. 2016 Oct 21;197(11):4283–91. doi: 10.4049/jimmunol.1601424 (PMC5114885; doi:10.4049/jimmunol.1601424)
Supplement: Data Supplement [file JI_1601424.zip › JI_1601424_Supplemental_Material_1.pdf]

Supplementary figure 1

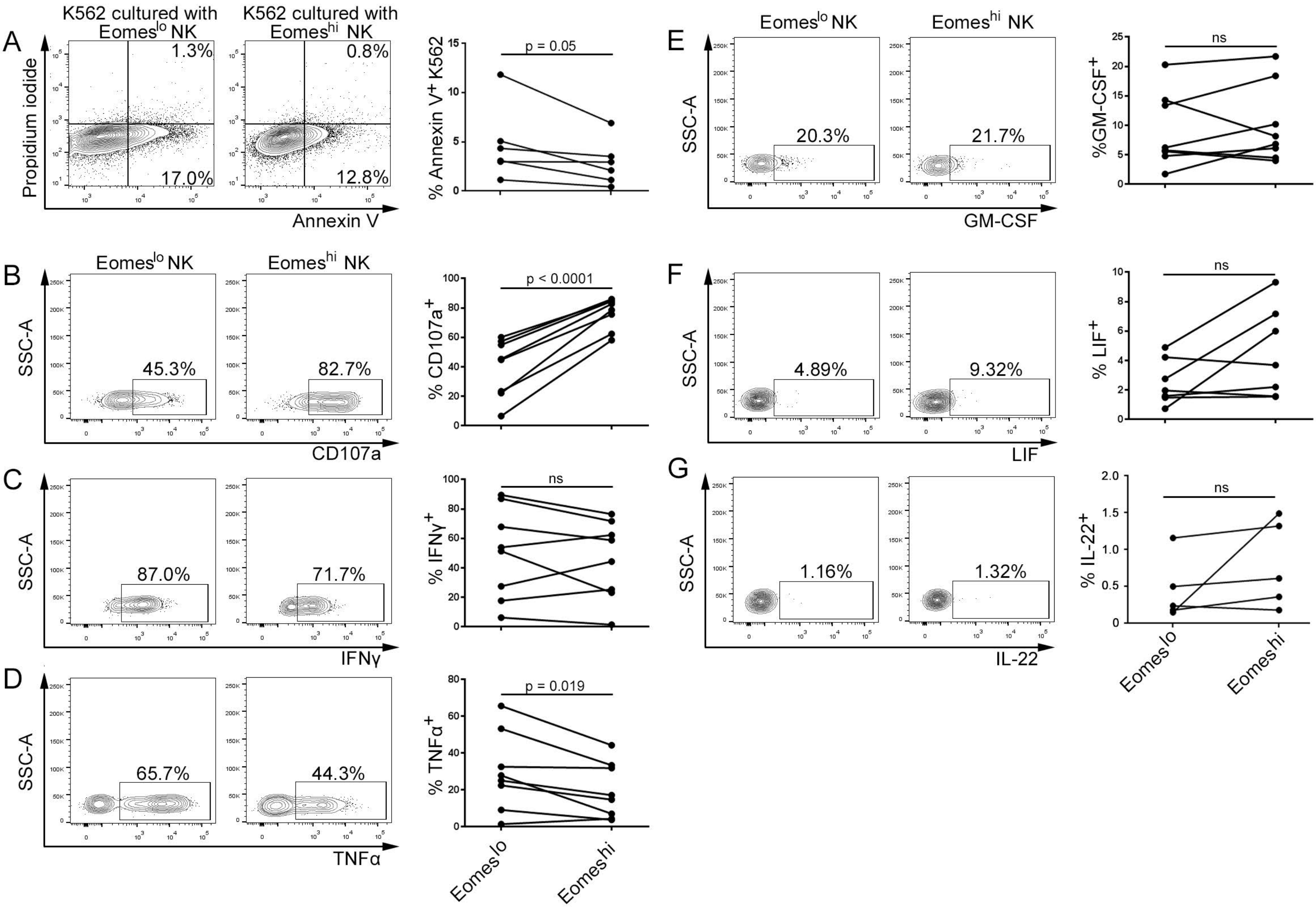

Supplementary figure 1: Cytotoxicity and cytokine production by liver NK cell subsets
A: Sorted Eomes^{lo} or Eomes^{hi} liver NK cells isolated from perfusion fluid were cultured with K562 cells for four hours. At the end of the culture period, K562 cell death was examined by Annexin V and propidium iodide staining. Example data from a single experiment is shown on the left, summary data from n = 6 experiments on the right, with baseline death in K562 alone subtracted from death seen in co-culture with NK cells. Significance was determined using a two-tailed Wilcoxon Signed Ranks test. B - G: Total liver leukocytes were cultured for four hours with PMA and ionomycin. CD107a (B) was included in the culture medium to assess degranulation. Intracellular staining was performed at the end of culture for IFNγ (C), TNFα (D), GM-CSF (E), LIF (F) and IL-22 (G) and the responses of Eomes^{lo} and Eomes^{hi} cells examined. Example staining from a single sample is shown on the left, summary data from n = 8 experiments (B - F) or n = 5 experiments (G) on the right. Significance was determined using two-tailed paired T tests.

Supplementary figure 2

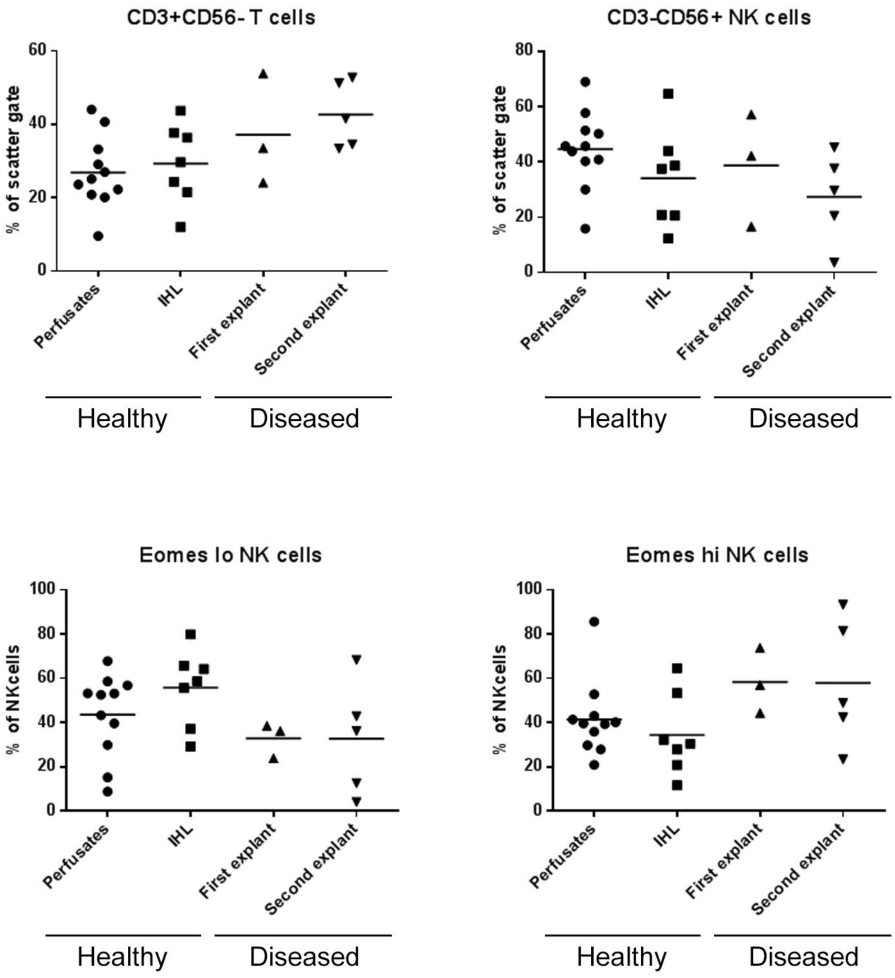

Supplementary figure 2: Immune cell composition in healthy and diseased livers
Fraction of CD3+ T cells, CD3- CD56+ NK cells, and Eomes lo and -hi NK cells as a proportion of total NK cells. Data are shown from: leukocytes isolated from perfusion fluid of healthy livers destined for transplant; intrahepatic leukocytes (IHL) from livers that were unsuitable for transplant because of vascular abnormalities, warm or cold ischaemic time or mild to moderate steatosis; livers explanted during transplant for NASH (first transplant only); livers explanted during retransplant for a variety of reasons (clinical details in Supplementary table 2).There were no significant differences between any of the groups (Kruskal-Wallis Test)

Figure	Transplant 1	Transplant 2	Transplant 2 – Transplant 1	Patient HLA	Transplant 1 donor HLA	Immunosuppressive medication prior to Transplant
2A-C	Primary sclerosing cholangitis	N/A	N/A	HLA-A2-; HLA-A3+	HLA-A2-; HLA-A3-	None given
2C	Primary sclerosing cholangitis	N/A	N/A	HLA-A2-; HLA-A3-	HLA-A2+; HLA-A3-	Tacrolimus 5mg bd
2C	Hepatitis C	N/A	N/A	HLA-A2+; HLA-A3-	HLA-A2-; HLA-A3-	None given
2C	NAFLD	N/A	N/A	HLA-A2-; HLA-A3-	HLA-A2+; HLA-A3-	None given
2C	ALD	N/A	N/A	HLA-A2+; HLA-A3-	HLA-A2-; HLA-A3+	None given
2C	Primary sclerosing cholangitis	N/A	N/A	HLA-A2-; HLA-A3-	HLA-A2-; HLA-A3+	None given
2C	Hepatitis C/HCC	N/A	N/A	HLA-A2-; HLA-A3+	HLA-A2+; HLA-A3-	None given
2D-F	Primary sclerosing cholangitis	N/A	N/A	HLA-A2-; HLA-A3-	HLA-A2+; HLA-A3+	None given
2F	ALD	N/A	N/A	HLA-A2+; HLA-A3-	HLA-A2-; HLA-A3-	None given
2F	Primary sclerosing cholangitis	N/A	N/A	HLA-A2-; HLA-A3-	HLA-A2+; HLA-A3-	Mercaptopurine 75mg
3A	Hepatitis C/HCC	Hepatic artery thrombosis	8 days	HLA-A2-; HLA-A3-	HLA-A2+; HLA-A3-	Azathioprine 75mg; prednisone 20mg; Tacrolimus 5mg bd;
3B	Primary sclerosing cholangitis	Chronic rejection	3 years	HLA-A2-; HLA-A3-	HLA-A2+; HLA-A3-	Prednisone 15mg;
3C	Primary sclerosing cholangitis	Ischaemic cholangiopathy	6 years	HLA-A2-; HLA-A3-	HLA-A2+; HLA-A3-	None given
3D	Hepatitis C	Hepatitis C	6 years	HLA-A2-; HLA-A3-	HLA-A2-; HLA-A3+	Mycophenolate mofetil 250 mg bd; Basililiximab 20 mg
3E	Primary sclerosing cholangitis	Ischaemic cholangiopathy	13 years	HLA-A2-; HLA-A3-	HLA-A2+; HLA-A3-	Tacrolimus 5mg bd

Supplementary table I: Clinical details for transplant patients. The right-most column shows immunosuppressive medication received by the patient on the day before transplant, but does not necessarily represent their usual daily medication (for example, basililiximab was given as a single dose on this day and some patients discontinued some of their medications while awaiting transplant). Non-immunosuppressive medications are not shown.
